# Immunity to the model intestinal helminth parasite *Heligmosomoides polygyrus*

**DOI:** 10.1007/s00281-012-0347-3

**Published:** 2012-10-11

**Authors:** Lisa A. Reynolds, Kara J. Filbey, Rick M. Maizels

**Affiliations:** 1 Institute of Immunology and Infection Research, University of Edinburgh, Edinburgh, EH9 3JT UK; 2 Institute of Immunology and Infection Research, University of Edinburgh, West Mains Road, Edinburgh, EH9 3JT UK

## Abstract

*Heligmosomoides polygyrus* is a natural intestinal parasite of mice, which offers an excellent model of the immunology of gastrointestinal helminth infections of humans and livestock. It is able to establish long-term chronic infections in many strains of mice, exerting potent immunomodulatory effects that dampen both protective immunity and bystander reactions to allergens and autoantigens. Immunity to the parasite develops naturally in some mouse strains and can be induced in others through immunization; while the mechanisms of protective immunity are not yet fully defined, both antibodies and a host cellular component are required, with strongest evidence for a role of alternatively activated macrophages. We discuss the balance between resistance and susceptibility in this model system and highlight new themes in innate and adaptive immunity, immunomodulation, and regulation of responsiveness in helminth infection.

## Introduction

### *Heligmosomoides polygyrus*: a model organism

Chronic helminth infections remain a huge global health problem, causing extensive morbidity in both humans and livestock. Many of the most prevalent helminth parasites are difficult to study in the laboratory, as they have co-evolved with, and are closely adapted to, their definitive host species. However, model organisms such as *Heligmosomoides polygyrus*, a natural mouse parasite, offer tractable and informative systems to explore the mechanisms of immunity and immune evasion in helminth infections [1, 2].

*H. polygyrus* (previously named *Nematospiroides dubius*) is an intestinal nematode parasite in wild mouse populations that has successfully been transferred to the laboratory. It is phylogenetically placed in the same Suborder, Trichostrongylina, as the ruminant parasites *Haemonchus contortus* and *Teladorsagia circumcincta* and within the same Order, Strongylida, as the human hookworm parasites *Ancylostoma duodenale* and *Necator americanus* [3]. *H. polygyrus* is an appropriate model of these chronic helminthiases, as primary infections can persist for many months in susceptible strains of mice.

**Fig. 1:**
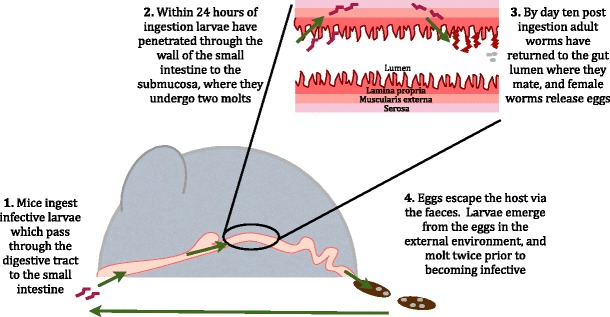
Lifecycle of *Heligmosomoides polygyrus* in mice

In an experimental setting, *H. polygyrus* is introduced by orally gavaging mice with infective L3 larvae. Following ingestion, within 24 h, larvae have penetrated through into the submucosa of the small intestine. Here they undergo two developmental molts, before emerging back into the lumen as adult worms, which feed on host intestinal tissue [4]. The adult worms coil around the small intestine villi to secure themselves, mate, and produce eggs, which are excreted in the feces. In the external environment, the eggs hatch and undergo two molts to become infective L3s, and so the lifecycle continues (Fig. 1).

The persistence of *H. polygyrus* within the murine host can be measured by determining the number of eggs that are released in the feces, or by enumerating adult worms remaining in the small intestine. As described below, the wide range of reagents available for assaying and manipulating the murine immune system cells in mice are being effectively applied to investigate responsiveness and immunity. The mechanisms behind helminth expulsion in mice can therefore be studied in order to make predictions about similar interactions between helminths and the immune system in livestock and humans, with a view to developing much-needed vaccines for control of these infections.

A further advantage of *H. polygyrus* is that the mammalian stages can be cultivated in vitro, where its secretory products, *H. polygyrus* excretory–secretory antigens (HES), can be collected, and individual components can be purified and identified [5]. This provides a fruitful strategy to test defined parasite molecules in vitro and in vivo for immunomodulatory functions and as candidate vaccine antigens.

## Genetics of susceptibility to *H. polygyrus*

In primary infections of different mouse strains, the length of time *H. polygyrus* can persist and the degree of response it provokes shows considerable variation, and some genotypes are also poor at rejecting challenge infections following immunisation.

Table 1 shows a summary of “responsiveness” to *H. polygyrus* in different mouse strains, based on adult worm survival and fecundity after primary and secondary infection. The genetic factors controlling strain differences in resistance to infection include the major histocompatibility complex (MHC) H-2 loci, with weak responders among the H-2^k^ and H-2^b^ genotypes and the H-2^q^ or H-2^s^ genotypes associated with a rapid response [6, 7].

**Table 1:** Strain-specific immunity to *H. polygyrus*

Responsiveness	Strain	Immune mechanisms investigated
Slow (>20 weeks to expel worms)	CBA	Primary response involves significantly lower cell numbers in the MLN than other strains [193, 194], very few mast cells in the gut [193], low levels of mMCP in serum and intestinal lavage [51, 53], and low eosinophilia [14]
	C3H	
	SL	
	A/J	
		Have no, or very weak, protective response to re-challenge [6, 53, 195]
Intermediate (8–20 weeks)	C57BL/6	C57BL/10 mice show less rapid and lower eosinophilia levels in circulation, after both primary *H. polygyrus* infection or injection of parasite antigens, than NIH mice [196]
	C57BL/10	
	129/J	
Fast (6–8 weeks)	DBA/2	NIH mice produced a higher peak of lymphocytosis, neutrophilia and monocytosis in the circulation than C57BL/10 mice after primary infection [197]
	BALB/c	
	NIH	
Rapid (4–6 weeks)	SJL	SJL and SWR have quicker and stronger antibody responses than other strains, involving stronger recognition of a larger number of antigens on a Western blot of HES [198] and adult homogenate [193], and higher titers of parasite-specific antibody of different isotypes in serum [51, 193, 198]
	SWR	Infected SWR MLN cells produced higher levels of IL-3, IL-4 and IL-9 after ConA stimulation than NIH and CBA [52]
		Both strains show early peaks of serum tumor necrosis factor alpha, mMCP-1, intestinal mast cells and goblet cells, which precede the expulsion of the worms [51, 53]

Experiments in H-2 congenic C57BL/10 mouse strains show that although establishment of *H. polygyrus* larvae is equal between all strains (shown by worm counts 2 weeks postinfection), by week 9, egg and adult worm numbers differ strikingly. Those with H-2^s^ and H-2^q^ haplotypes expelled the parasites more rapidly [7, 8], while mice carrying H-2^b^ or H-2^k^ haplotypes backcrossed into the fast-responding BALB/c background were unable to expel worms quickly [7]. Resistance was shown to be conferred by more than one gene, as F1 hybrids of fast responders, SJL and SWR, display heightened abilities to expel worms, and is inherited in a dominant fashion as C57BL/10xSJL hybrids are as rapid in expulsion as the SJL parental strain [7, 9].

More recently, a study mapping quantitative trait loci in fast responding (SWR, H-2^q^) versus slow responding (CBA, H-2^k^) strains found significant effects on resistance to *H. polygyrus* trickle infection from positions on chromosomes 1, 2, 13, and 17 [10]. Several candidate resistance genes were identified, including as expected MHC (on chromosome 17), and also interleukin-9 (IL-9; on chromosome 13), both of which correlate with worm expulsion [10].

A notable gender bias in susceptibility is also observed, with female mice of all strains clearing primary infections faster than their male counterparts as also apparent to a lesser degree following secondary exposure [11–13]. The greater susceptibility of male mice correlates with higher fecundity of worms recovered from male hosts and a larger adult worm body size [14], indicating that the parasites are fitter than those from a female host.

Concurrent pregnancy and worm infection imposes increased physiological demands on the mother in terms of the energy required to fight infection and to nourish the fetus. This can lead to immunosuppression (diminished Th2 responses) [15] and adverse reproductive outcomes (small pup size) [15, 16]. The effects of pregnancy on maternal serum cytokines during *H. polygyrus* infection include increased levels of IL-1β and IL-6 at day 20 postinfection [15] and lower concentrations of IL-4, IL-5, IL-13, and mucosal mast cell protease (mMCP-1) [15]. Pregnant mice also show a small but significant increase in adult worm burdens [15].

## Models of resistance

Immunity to *H. polygyrus* can be studied in three separate settings each with distinct implications for human infection, namely, genetically determined, drug-induced, and vaccine-elicited immunity. In each of these contexts, the availability of numerous gene-targeted mouse strains and immunological reagents are being used to define immune system components and parameters required for immunity to infection.

As stated above, the outcome of primary *H. polygyrus* infection is strongly influenced by the genetic background of mice, with strains differing in their susceptibility to chronic infection. Studying how the immune response differs between those strains that endure chronic infections and those that are able to clear a primary infection has been highly instructive in defining the immune mechanisms the host must promote in order to clear the parasite.

If primary infection with *H. polygyrus* is cleared using antihelmintic drugs such as pyrantel pamoate or ivermectin [17], most mouse strains display a highly effective memory response, which provides immunity to reinfection [18]. Genetic background also impacts on resistance to reinfection, as BALB/c mice display significantly lower worm numbers postchallenge compared to C57BL/6 [19, 20].

Finally, HES administered in alum adjuvant has been shown to induce sterilizing immunity to *H. polygyrus* infection [21], and studies are ongoing to identify the specific components of HES and the immune mechanisms critical for this immunity.

### Challenge infection/Trickle infection

Although most laboratory studies employ a single bolus infection, doses used are far from physiological or representative of field conditions. Hence, some investigators have developed trickle infection regimes, for example, administering twice weekly low doses of infective larvae. Under these conditions, different mouse strains show a gradation of resistance patterns similar to those seen with single-bolus primary infection, in that NIH and SWR strains resolve infection (showing an initial increase in adult worm burden, a period of stability, and then finally expulsion) while CBA and C57BL/10 mice continue to accumulate increasing adult worm burdens over the course of repeated infections [22].

### Variation and adaptation by *H. polygyrus*

The strain of *H. polygyrus* used in laboratories worldwide is thought to have been isolated from wild Californian mice in the 1940s [23] and was known for some years as *Nematospiroides dubius*. The vast majority of the literature describing experiments with this isolate refers to the parasite as *H. polygyrus*. It was, however, suggested that this laboratory strain should be referred to as *H. polygyrus bakeri*, to differentiate it from wild strains of the parasite, considered to be *H. polygyrus polygyrus* (found in the wood mouse *Apodemus sylvaticus* in Europe), *H. polygyrus corsicus* (from the house mouse *Mus musculus* in Corsica) and *H. polygyrus americanus* (from the vole *Phenacomys intermedius* in North America) [24]. More recently, there has been an additional proposal of a name change for the laboratory isolate to *H. bakeri* [23], based on sequence divergence between laboratory and European wood mouse isolates [25]. This proposal has not received widespread support due to the preliminary nature of the data, the sequence variation even within the laboratory strain, and the need to remain consistent with previous literature [26]. Here, we refer to the laboratory strain of the parasite as *H. polygyrus*.

In proteomic studies on *H. polygyrus* secreted antigens (see below), extensive sequence variation was observed in some gene families [5], indicating that despite many years of laboratory propagation, the parasite strain remains highly polymorphic. Moreover, there are indications that antigen expression by *H. polygyrus* may vary or adapt according to the host strain of mouse [27, 28], with proteomic differences in adult worms recovered 4 weeks postinfection in either C57BL/10 (slow responder) or SWR (fast responder) hosts [28]. Phosphatidylethanolamine-binding protein and several nematode globins are overexpressed in worms from C57BL/10 compared to worms from SWR mice, and myosin, troponin, actin, and several unidentified proteins are overexpressed in the worms from fast responder mice compared to slow [28]. Differential expression of worm products in different host strains may shed light on pathways targeted by the immune system that impact on worm survival or death.

## Host immune responses

The critical requirement for the adaptive immune response in control of the parasite is illustrated when B- and T-cell responses are lacking. Severe combined immunodeficient (SCID; B- and T-cell deficient) and athymic mice show impaired expulsion of adult worms, maintaining high worm burdens several weeks post infection by which stage wild-type counterparts had expelled the majority of their worms [29, 30]. Treatment with anti-CD4 results in higher fecundity of female worms in a primary infection [31] and transfer of the effector T-cell subset from chronically infected animals significantly reduced worm burdens when transferred to naive mice before infection [32]. Although B-cell deficiency does not affect the outcome of primary *H. polygyrus* infection, B-cell or antibody deficiency significantly compromises the ability to expel a secondary challenge infection [33].

### T-cell responses

*H. polygyrus* infection induces a strongly polarized Th2 response, which has been shown to be critical in control and expulsion of the worm [31]. A primary *H. polygyrus* infection induces IL-3, IL-4, IL-5, and IL-9 gene expression in the mesenteric lymph nodes (MLN) and Peyer’s patches [34] and elicits the release of high concentrations of IL-4, IL-5, IL-9, IL-10, and IL-13 protein from MLN, spleen, and lamina propria mononuclear cells (LPMC) cultured with parasite antigens [32, 35, 36]. IL-4 is the most critical single cytokine for protection against primary and secondary *H. polygyrus* infection (both in expulsion of adult worms and inhibiting their egg production) [31]. Immunity to secondary infection is diminished by blocking antibody to IL-4 but completely abolished when the IL-4R is also blocked [31]. This suggests that IL-13, which also signals through IL-4Rα, can partially compensate for the loss of IL-4, but in the absence of signaling from both cytokines, protection against reinfection with *H. polygyrus* is lost. Blocking of IL-5 by antibody treatment had no effect on worm expulsion [31]. When IL-4 is administered as a complex with anti-IL-4 (IL-4C) to extend the activity time of this cytokine, wild-type BALB/c mice expelled *H. polygyrus* more rapidly [29]. This effect did not depend on the adaptive immune system, as *H. polygyrus* expulsion was also seen in SCID mice and anti-CD4-treated BALB/c mice, which were given the IL-4 complex [29]. After primary infection, CD4^+^IL-4^+^ T cells disseminate around the body to lymphoid and nonlymphoid organs, such as airways, peritoneal cavity, and liver, and have a lower apoptotic potential [37, 38]. These findings may be illustrative of peripheral reservoirs of long-lived memory Th2 cells primed to respond to subsequent infection challenges by the worm.

Further studies have delineated the costimulatory signals required to mount Th2 responses to *H. polygyrus* infection. By blocking signaling through both CD80 (B7-1) and CD86 (B7-2) with specific antibodies or a CTLA4-Ig construct, IL-4 expression, Th2 expansion, and IgE production in response to *H. polygyrus* were ablated [39, 40]; interestingly, an innate IL-5 response remained intact when T-cell costimulation was inhibited. Blocking antibodies against CD80 or CD86 alone had little effect [40], and while early (day 6 postinfection) responses to *H. polygyrus* were unaltered in CD86-deficient mice [41], by day 14 postinfection, CD86-deficient mice had higher parasite egg burdens and decreased Th2 responses [41]. This showed that, while CD86 was not required for the initiation of the antiworm response, it was necessary for its progression and persistence.

Although both CD80 and CD86 normally ligate to T-cell CD28, CD28-deficient mice were found to have no impairment in the early CD4^+^IL-4^+^ response, indicating an alternative mechanism for Th2 costimulation in *H. polygyrus* infection [42]. Moreover, while the primary T-cell response to infection is CD80/CD86-dependent as outlined above, on secondary infection, memory helper T cells do not require CD80 or CD86 costimulation for their activation to protect against challenge [43, 44]. Studies into an additional costimulatory molecule, OX40L (CD143), showed that it is specifically required to promote IL-4 production from T cells (and the associated rise in IgE), without affecting Th2-cell expansion, migration, germinal center formation, or IgG1 levels [45].

T-follicular helper cells are now recognized as the instrumental subset, which induces germinal center formation and isotype switching in B cells, by migrating to B-cell follicles and releasing cytokines including IL-21 [46], while also producing IL-4 in the MLN of *H. polygyrus*-infected mice [47]. IL-21 plays key roles by stimulating multiple cell types across a range of infections [48]. In *H. polygyrus* infection, IL-21 deficiency results in reduced intestinal granuloma formation, impaired T-cell expansion and survival, and lower numbers of circulating basophils and eosinophils [49]. IL-21 also provides a critical signal for the differentiation of B cells into plasma cells and for protection against secondary challenge infection with *H. polygyrus* [50].

### B-cell and humoral responses

In general, the intensity and speed of parasite-specific antibody responses are greater in more resistant mouse strains such as SJL and SWR than in susceptible strains. Specifically, the IgG1 and IgE responses (to adult worm homogenate and HES) negatively correlate across strains with worm survival after a primary infection [12, 51, 52]. However, after repeated low-dose (“trickle”) infections, there was little difference between slow and fast responder strains in any antibody isotype measured to larval and worm antigens [53].

B cells, as well as secreting antibodies, also produce cytokines and costimulatory molecules that promote and amplify the T-cell response in a selective manner [54, 55]. Interestingly, the greatest increase in cell number in MLN after *H. polygyrus* infection is in the B-cell compartment [32, 56].

The protective response to secondary challenge with *H. polygyrus* is dependent on B cells, as μMT and JHD mice (both of which lack B cells) cannot clear the parasites [57–59]. Defective immunity in B-cell-deficient mice is not due to an impairment of Th2 responses, or to T regulatory cell (Treg) activation, development, or differentiation, as a pronounced local Th2 response in the intestinal tissues occurred with or without B cells, in both primary and secondary infection [58, 59]. However, a separate study showed impairment of the Th2 response in B-cell deficient mice, with significantly lower T-cell expansion and cytokine production [57]. These authors showed that a sufficient T-cell memory response was B-cell dependent and that immunity required B cells to produce the cytokines IL-2 and tumor necrosis factor alpha [57]. This discrepancy remains unclear, and B cells seem to have differing roles in other helminth infections. During a primary infection with the colon-residing murine nematode parasite *Trichuris muris*, B cells are required for resistance and the development of a Th2 response [60]; however, in primary and secondary infection with another gastrointestinal nematode, *Nippostrongylus brasiliensis*, Th2 responses and worm expulsion are B-cell independent [58].

The specific role of antibody in mediating protection against *H. polygyrus* has also been investigated using mice with targeted deficiencies within the B-cell compartment. For example, μs mice have secretory IgM-deficient B cells, but can produce parasite-specific class-switched IgG1 and IgE, and are able to clear secondary infection [57]. However, when crossed with a null activation-induced deaminase transgene, the resultant mice are unable to undergo affinity maturation or secrete antibody of any isotype and are not protected from secondary challenge with the parasite [57]. Using selective isotype knockout mice given a secondary *H. polygyrus* infection, it was found that IgE had no role in protection, and IgA had a minor role, leaving IgG as the major class-switched isotype leading to protection [59]. Indeed, it has been long known that the humoral response is dominated by parasite-specific IgG1 and that serum fractions with highest parasite-specific IgG1 activity afford greater protection when transferred to an infected animal [61]. Even transfer of whole serum from immune wild-type donors to JHD-recipient mice can significantly reduce the number of adult worms left in the intestine after a challenge infection [58].

Primary *H. polygyrus* infection also elicits an extraordinary increase in nonspecific serum IgG1 levels (hypergammaglobulinemia) [59, 61, 62], and following repeated trickle infections over 4 weeks, serum IgG1 concentrations can reach 30 times the normal level seen in uninfected mice [62, 63]. Despite these high concentrations, transfer of serum from 28-day infected mice does not protect naive animals from infection [59, 64]. In contrast, serum raised after multiple *H. polygyrus* infections is protective against adult worm survival when transferred into naive recipients [64, 65], presumably reflecting the higher ratio of parasite-specific to nonspecific IgG following repeated infection. The mechanisms through which such high levels of polyclonal IgG1 are produced in response to *H. polygyrus* (and other parasitic worms [63]) remain to be explored.

### Innate immune responses

Innate immune cells are the initial responders to a *H. polygyrus* infection and are also implicated in the end-stage expulsion of parasites. Innate cells release type 2 cytokines that can act directly to alter gut physiology and polarize the adaptive immune response, while themselves employing helminth-damaging or killing mechanisms [66, 67].

### Dendritic cells

Dendritic cells (DCs) are the predominant innate antigen-presenting cell that are required to prime Th2 responses against helminths [68]. DCs loaded with helminth products in vitro can be transferred to naive animals to induce a Th2 response [69] and have been shown to inhibit allergic airway inflammation when transferred from a helminth-infected animal, resulting in increased numbers of Tregs and a downregulation of Th2-mediated inflammation [70]. When CD11c^+^ DCs are depleted using CD11c.DTR mice [71] that coexpress CD11c with the human diphtheria toxin receptor (DTR), a Th2 response against several helminths (including *H. polygyrus*) is severely compromised [72, 73].

### Macrophages

The alternative activation of macrophages is a hallmark of helminth-elicited Th2 responses and is associated with high expression of a characteristic set of gene products, including Ym1, RELM-α (FIZZ-1), arginase-1, IL-4Rα, and the mannose receptor CD206 [66, 74]. Macrophages can differentially express the enzymes nitric oxide synthase 2 (NOS-2) and arginase-1, which compete for the common substrate l-arginine, and are competitively induced by interferon gamma (IFN-γ) and Th2 cytokines (IL-4, IL-10, IL-13, and IL-21), respectively [75–78]. The activation state of macrophages in helminth infections is sufficiently plastic to respond to changing stimuli, as helminth-induced alternatively activated macrophages restimulated ex vivo with lipopolysaccharide and IFN-γ switch to a classically activated phenotype [79], suggesting that such plasticity may also occur in vivo.

Alternatively activated macrophages are critical to the protective immune response to secondary *H. polygyrus* infection, as mice lost the ability to reject challenge infections when depleted of macrophages via clodronate treatment, or when treated with *S*-(2-boronoethyl)-l-cysteine (BEC), a pharmaceutical inhibitor of arginase [80]. Arginase-1 may directly harm parasites, as *H. polygyrus* exhibited higher levels of cytochrome oxidase, a marker of a stress response, in a secondary infection compared to a primary infection, and this increase was lost following BEC administration [80]. In contrast to arginase, no antiparasite function has been found for Ym1, a member of the chitinase-like family of proteins that lacks demonstrable chitinase activity [81]. Ym1 does bind heparin on cell surfaces and in the extracellular matrix [81], which may indicate a role for Ym1 and alternatively activated macrophages in mediating repair of tissue damage caused by *H. polygyrus* when migrating through the intestinal wall [82].

Alternatively activated macrophages may also be important mediators of the smooth muscle hypercontractility response to intestinal helminth infections, at least in the context of a *N. brasiliensis* infection, as depleting macrophages via clodronate-treatment blocked smooth muscle hyperactivity and impaired worm expulsion [83].

### Neutrophils

Perhaps surprisingly, the role of the principal granulocyte cell types (neutrophils, eosinophils, and basophils) has not been directly evaluated in *H. polygyrus* infection. Neutrophils are prominent in primary and, to a lesser extent, secondary granulomas during a *H. polygyrus* infection [80, 84, 85]. The finding that neutrophils are less prevalent in a setting of heightened resistance may indicate that they are not a key cell type in immunity. To date, a protective function for neutrophils during helminth infections has only been reported for infections of mice with tissue-migrating larvae of the human nematode parasite *Strongyloides stercoralis*, although even in this case killing was more effective when eosinophils were present alongside neutrophils [86, 87].

### Eosinophils

No role for eosinophils in *H. polygyrus* expulsion has yet been described. In a genetic model of eosinophil deficiency, in which an eosinophil-specific site in the GATA-1 promoter is deleted [88], mice showed impaired resistance to challenge infections with *N. brasiliensis* [89]; significantly, in the absence of eosinophils, greater numbers of tissue larvae migrated to the lung, but expulsion of those parasites that subsequently reached the gut was unimpaired in the eosinophil-deficient mice. Eosinophilia in response to *N. brasiliensis* infection was blocked when mice were administered anti-IL-5 antibody [90, 91], but this had no impact on adult worm recovery [90], providing additional evidence that eosinophils are not a critical mediator of expulsion in this system. Anti-IL-5 treatment during *H. polygyrus* infection also had no impact on worm burden [92], and eosinophils within the gut wall have been reported to be inhibited during *H. polygyrus* infection in a manner reversible with anti-transforming growth factor beta (anti-TGF-β) antibody treatment [93].

### Basophils

As with the other granulocytes, few studies have investigated the role of basophils in *H. polygyrus* infections. In other gastrointestinal nematode infections, basophilia is conspicuous, and their presence may be required for optimal *N. brasiliensis* expulsion [94]. Worm expulsion of *T. muris* was impaired when basophil numbers were depleted using MAR-1 antibody [95]; however, this antibody targets the FcεRI, which is also expressed by mast cells, so this does not conclusively prove a role for basophils alone.

### Mast cells

Mast cells are major players in the intestinal immune response to infection with *H. polygyrus*, as expulsion correlates with epithelial mastocytosis [52, 96] and elevated intestinal fluid levels of mMCP-1 in different murine strains [51]. Mast cells may promote helminth damage by increasing the permeability of the gut via mMCP-1-mediated breakdown of epithelial tight junction proteins [97, 98], thereby increasing luminal flow and disrupting the niche of parasitic helminths. Increased permeability of the gut in response to *Trichinella spiralis* is blocked in mMCP-1-deficient mice, which were less effective at clearing the worms than wild-type counterparts [98]. The mast cell response to *T. spiralis* (and *N. brasiliensis*) infection, however, is ablated in mice carrying an *H. polygyrus* coinfection [99] arguing that the latter parasite is able to suppress host mastocytosis to a significant degree.

Most in vivo studies on mast cells in helminth infection have involved the mast-cell-deficient mice *Kit* ^*W*^ */Kit* ^*W-v*^ which carry a mutated gene encoding the tyrosine kinase receptor c-kit. During *H. polygyrus* infections, these mice produce higher egg numbers than wild-type controls, indicative of impaired immunity [100]. Consistent with this, reduced egg production was seen in Tg2Rbeta mice [101], which exhibit mastocytosis.

In terms of protective immunity to adult worms, however, *Kit* ^*W*^ */Kit* ^*W-v*^ mice were found to be similar to wild type in slowly expelling primary *H. polygyrus* infection between 4 and 9 weeks of infection [100]. However, a more recent report has that shown *Kit* ^*W*^ */Kit* ^*W-v*^ mice and another mast cell deficient strain, *Kit* ^*W-sh*^ mice, do have impairments in *H. polygyrus* expulsion, as both strains had higher worm burdens than wild-type mice after 3 weeks of a primary *H. polygyrus* infection [102]. The same authors also showed that *Kit* ^*W*^ */Kit* ^*W-v*^ mice were not resistant to a secondary *H. polygyrus* infection, yet control wild-type mice were able to clear the infection [102]. The reason for the discrepancy between the reports on the ability of *Kit* ^*W*^ */Kit* ^*W-v*^ mice to clear *H. polygyrus* is not clear, and more studies are required to confirm the importance of mast cells during infections. If mast cells do contribute to expulsion of *H. polygyrus*, it could be via their contributions towards priming a Th2 response early in infection, as well as their potential role as a later effector cell. *Kit* ^*W*^ */Kit* ^*W-v*^ MLN cells did not show the high levels of *H. polygyrus*-antigen-specific Th2 cytokines produced by wild-type MLN cells in response to *H. polygyrus* [102].

It should be noted that both *Kit* ^*W*^ */Kit* ^*W-v*^ and *Kit* ^*W-sh*^ mice have defects that extend beyond a mast cell deficiency [103]. Many of the recently described subsets of lineage negative innate type 2 cells, discussed below, express c-kit, and so it is likely that some of the deficiencies of *Kit* ^*W*^ */Kit* ^*W-v*^ and *Kit* ^*W-sh*^ mice can be explained by the additional disruption of these cell types.

### Innate lymphoid cells

Recently, a number of studies have identified a population of lineage marker negative innate lymphoid cells (ILC), which produce type 2 cytokines (particularly IL-5 and IL-13) in response to epithelial cell-derived cytokines, including IL-25, IL-33, and thymic stromal lymphopoietin (reviewed in [104]). IL-25 may also be derived from other cell types, such as mast cells [105], but the importance of IL-25 from this source is as yet unknown. Epithelial cells produce elevated levels of these cytokines in response to damage, thereby raising the first alarm leading to Th2 responses (reviewed in [106]). Trefoil factor 2 (TFF2) is a molecule involved in epithelial cell repair, which induces IL-33 production by epithelial cells in response to damage caused by *N. brasiliensis* [107]. TFF2^−/−^ mice did not show the elevated epithelial IL-33 levels in response to *N. brasiliensis* that wild-type mice did, instead having lower serum IL-4 levels after 7 days of infection, and delayed worm expulsion [107]. Similarly, Th2 cytokine production is delayed, and *N. brasiliensis* expulsion is impaired in IL-25^−/−^ mice, which correlates with the absence of a non-B non-T-cell c-kit^+^ IL-4, IL-5, and IL-13 producing population induced in infected wild-type mice or mice administered rIL-25 or rIL-33 [108–110]. A role for ILCs has not yet been reported during a *H. polygyrus* infection though it seems likely that these cell types are important inducers of Th2 responses during all intestinal helminth infections.

### Gut physiology and intestinal epithelial cell function

IL-4 and IL-13, derived from innate or adaptive sources, are likely to have direct effects on the physiology of the gut as well as on effector cells that promote helminth expulsion. Although few changes in epithelial cell function are noted during primary *H. polygyrus* infection, in secondary infections, increased mucosal permeability, decreased ion absorption, and increased prosecretory effects in response to prostaglandin E2 and histamine were seen [111, 112]. Moreover, these changes were dependent on the IL-4R and STAT6 and were reproduced by IL-4C administration [111, 112]. These alterations to the worm’s environment may interfere with its abilities to feed on the intestinal tissue [4] or remain wrapped around the villi in the small intestine.

### Goblet cell function

Within the intestinal epithelial layer are specialized goblet cells that secrete innate defence proteins as well as large quantities of mucins, the key components of mucus. Goblet cell hyperplasia develops in response to intestinal helminth infections, including *H. polygyrus*, where hyperplasia is dependent on a functional T-cell response [30].

Enhanced mucus production has been suggested to act against helminth establishment, and it may be that specific components within the mucus play a role in control of helminth expulsion. RELM-β (FIZZ-2) is a cysteine-rich mediator expressed by goblet cells in response to IL-13 and is important for the normal control of epithelial cell barrier permeability [113, 114]. RELM-β^−/−^ mice do not expel a secondary *H. polygyrus* infection as rapidly as wild-type mice and adult *H. polygyrus* worms treated in vitro with recombinant RELM-β prior to transfer to a new host survived less well than untreated adult worms [115]. This suggests that RELM-β is an important factor in inhibiting worm survival, perhaps by interfering with worm chemotaxis and nutrition [113, 115]. Secretion of MUC2, a major component of mucus in both the small and large intestine, is also upregulated during a *H. polygyrus* infection [30]. No evidence for a role for MUC2 in expulsion of *H. polygyrus* has yet been reported; however, MUC2 production correlates with the expulsion of *T. muris* [116, 117] and *N. brasiliensis* [118].

### Smooth muscle contraction

Both IL-4 and IL-13 enhance smooth muscle contractility in the small intestine [119], a mechanism that has been shown to be important for resistance to other helminth infections including *Schistosoma mansoni* [120], *T. spiralis* [121, 122], and *N. brasiliensis* [123]. Increased intestinal smooth muscle contractility has been shown after infection with *H. polygyrus* [124]. Both *N. brasiliensis* and *H. polygyrus* infections cause an upregulation of protease-activated receptor (PAR)_2_ messenger RNA in the small intestine, and a PAR_2_ agonist caused smooth muscle contractility, which was enhanced in both parasite-infected groups and, for *N. brasiliensis* at least, was dependent on STAT6 [124]. The infection-induced hypercontractility in the presence of PAR_2_ agonist was lost when nerve conduction was blocked using the neurotoxin TXX [124]. Whether smooth muscle hypercontractility plays a critical role in *H. polygyrus* expulsion has yet to be determined.

### Granuloma formation

A striking phenomenon in infection is the formation of granulomas around the site of larval invasion in the intestinal tract, and they are more numerous in resistant strains of mice [125], particularly following secondary *H. polygyrus* infection. While granuloma formation is Th2 dependent, their function has yet to be determined, either in damaging larval worms encysted in the submucosal layer of the small intestine or in tissue repair after *H. polygyrus* has departed into the lumen of the gut [126]. Granulomas in both primary and secondary infection consist of neutrophils, macrophages, dendritic cells, and eosinophils; in secondary infection, CD4^+^ Th2 cells and a high proportion of alternatively activated macrophages rapidly migrate to the site of infection to surround the larvae [80, 84, 85].

## Immuno-regulatory cells in chronic infection

### Regulatory T cells

Several categories of T cells exert suppressive or immunomodulatory effects, most prominently the subset of Tregs expressing the transcription factor Foxp3. Sustained expression of Foxp3 is required to maintain Treg suppressive function, as in its absence Tregs acquire effector T-cell functions [127], and conversely, the forced expression of Foxp3 confers suppressor function to CD4^+^CD25^−^ T cells [128]. Tregs are essential during infection to protect against immune-mediated pathology while still allowing a sufficiently robust response to clear the pathogen [129]. Indeed, when Foxp3^+^ T cells are removed at early stages of an infection with *H. polygyrus*, pathology of the small intestine is significantly worse, with higher numbers of effector T cells, IL-4, and IL-13 [130].

Foxp3 is constitutively expressed in a subset of regulatory cells termed natural Tregs, but expression can also be induced in resting Foxp3^–^ peripheral T cells. Natural Tregs develop in the thymus to limit autoreactive T cells, while inducible Tregs leave the thymus as conventional T cells and are converted through TGF-β, IL-10, and retinoic acid stimulation [129]. Treg induction is particularly favored in the intestine and gut-associated lymphoid tissues, where *H. polygyrus* resides and where TGF-β is highly enriched [32, 131]. Tregs, which express the integrin CD103 (CD4^+^CD25^+^CD103^+^), are more suppressive of CD4^+^ effector cells in vitro and release significantly more IL-10 into culture supernatants after stimulation with *H. polygyrus* primed dendritic cells than CD4^+^CD25^+^CD103^−^ Tregs [32].

A strong Treg response develops in the MLN and spleen of *H. polygyrus*-infected mice, peaking at day 28 postinfection [35]. CD25^+^CD103^+^ cells are the subset in the CD4^+^ compartment that shows the greatest increase in cell number (compared to CD25^−^CD103^−^ effector cells and CD25^+^CD103^−^ cells) [32, 35]. Most significantly, Foxp3 can also be induced in naive T cells by HES in vitro, in a manner analogous to TGF-β, due to parasite-derived TGF-β-like activity [132] (discussed below). Inhibition of TGF-β signaling during *H. polygyrus* infection using the inhibitor SB431542 reduces adult worm burden and results in an increased Th2 response [132], while administration of anti-TGF-β neutralizing antibody has also been reported to result in lower worm numbers [93]. When TGF-β signaling is lost only on CD4^+^ T cells, in TGF-βRII DN mice [133], there was no reduction in adult *H. polygyrus* burden compared to wild-type mice; in fact, *H. polygyrus* is more fecund [134, 135]. This is likely due to excessive IFN-γ production in the absence of CD4^+^ TGF-β signaling, as when IFN-γ-deficient TGF-βRII DN mice were infected with *H. polygyrus*, fewer adult worms survived after 28 days than in IFN-γ-sufficient TGF-βRII DN mice [134], illustrating the importance of both TGF-β and IFN-γ in determining susceptibility to *H. polygyrus*.

*H. polygyrus* infection also induces CD8^+^ Tregs in the lamina propria of the small intestine, which can inhibit T-cell proliferation in vitro in an IL-10 and TGF-β independent manner [36, 136].

### Regulatory B cells

In addition to Tregs, regulatory B cells (Bregs) have also been described that produce IL-10 and TGF-β, and can dampen potentially harmful immune responses [137]. Bregs induced during helminth infections can not only downregulate pathology elicited by schistosome eggs [138] but also ameliorate immunopathologies such as multiple sclerosis [139] and anaphylaxis [140] in humans and mice. While the role of Bregs in parasite persistence has not been directly investigated in *H. polygyrus* infection, suppressive B cells expand in the MLN of infected C57BL/6 mice, which on transfer to uninfected hosts, suppress airway allergy and inflammation in experimental autoimmune encephalomyelitis [56].

### Proregulatory DCs

Different subsets of DCs have been identified, which are markedly altered during helminth infection. In the MLN of *H. polygyrus*-infected mice, the proportion of CD11c^high^CD8α^intermediate^ DCs declines in infection, indicating a reduced migration of cells from the lamina propria [141]. Moreover, there is a sharp increase in the proportion of tolerogenic CD11c^lo^ DCs in the MLN; this cell type responds suboptimally to Toll-like receptor stimulation, is unable to prime a strong Th2 response from T cells, but induces much higher proportions of CD4^+^CD25^−^ to express Foxp3 than the conventional CD11c^hi^ subset [142, 143].

This effect was mirrored in an in vitro setting when OVA-pulsed bone marrow-derived DCs (BMDCs) were cultured with HES and showed lower costimulatory molecule expression and cytokine output compared to untreated OVA-pulsed BMDCs [144]. These cells also induced IL-10-secreting CD4^+^CD25^+^Foxp3^+^ cell generation from CD4^+^ cocultures [144], indicating a potential regulatory pathway initiated by *H. polygyrus* products.

## Vaccine-induced immunity

Irradiated *H. polygyrus* larvae given orally stimulate protection against subsequent challenge [62, 145–148]. Notably, the efficacy of this irradiated larval vaccine is diminished by the coadministration of unirradiated larvae, indicating that the development of adult worms is able to inhibit development and/or expression of protective immunity against subsequent reinfection [146, 147]. The ability of adult worms to suppress protective immunity was further demonstrated by vaccine failure in mice given irradiated larvae before or after receiving adult parasites by intraintestinal laparatomy [146] or oral gavage [149].

As well as infective (L3) stage larvae, live L4 larvae isolated on days 4 or 6 postinfection from the intestinal wall of donor-infected animals given subcutaneously, elicit an ~95–100 % reduction in worms present 3 weeks after challenge compared to unimmunized controls [150]. When immunization was performed with late-stage L3 (isolated 2 days postinfection) or L5 larvae (isolated 8 days postinfection) a lower degree of protective immunity was induced (~60 and ~70 % reduction respectively compared to unimmunized controls) [150].

Recently, an effective nonliving vaccine against *H. polygyrus* has been developed, in the form of total HES administered with alum adjuvant, which induced sterile immunity against infection [21]. Earlier work had shown that mice immunized with a 60,000 mol wt HES-derived glycoprotein isolated from HES prior to infection had lower egg burdens than control mice, indicating an antifecundity effect of vaccination with this component [151].

## Molecular basis of chronic infection

### Parasite excretory–secretory products

The ability of helminth parasites to persist in the host for many months or years, evading host immunity, is most likely due to the secretion of active immunomodulatory molecules [152]. The secretome of a parasite is likely to continually mediate interactions with the host, through direct contact with host cells in proximity to the worm, and potentially systemically. Helminth-secreted immunomodulators have been intensely studied, with some candidates now being tested for treatments of other diseases and as targets for antiparasite drugs [153].

Early investigation of HES found immunomodulatory factors that suppressed proliferation of mitogen-stimulated lymphocytes [154] and Th2-dependent antibody production to a bystander antigen through effects on T cells [155]. More recently, HES has been shown to display a wide range of immunomodulatory activities, including inhibiting activation of DCs [144, 156], induction of Tregs [132], and suppression of airway allergic inflammation [157].

The individual components of the complex HES mixture from adult worms have recently been defined through proteomic and sequencing technology with the identification of several hundred proteins in HES [5, 158]. Most prominent and numerous among the HES products are >20 members of the Venom allergen/*Ancylostoma* secreted protein-like (VAL) multigene family, which show extensive sequence variation between genes [5]. VALs have also been found to be highly immunodominant as indicated by recognition of primary and secondary infection serum, and monoclonal antibodies [21], although their function is as yet undefined [5, 158]. These studies also found acetylcholinesterases and proteases to be abundant in HES [5] along with apyrases, lipid-binding proteins, lysosymes, globins, and vitellogenin homologues [5, 158].

### Stage and sex specificity of HES

A small number of studies have identified lifecycle-stage-specific expression patterns of certain HES antigens. Infective larval stages of *H. polygyrus* secreted the highest levels of proteolytic enzymes and acetylcholinesterase [159, 160], which may be involved in migration through host tissues directly after infection. Calreticulin has been shown to be highly expressed in L4 larval stages and is localized in areas associated with excretory–secretory processes [161]. A TGF-β homologue has been shown to be abundantly expressed in adults compared to larval stages, which may indicate an immunomodulatory function when the adults reside in the lumen of the intestine for long periods of time [162]. A limited number of sex-specific adult antigens in both HES and on the cuticle of the worms have also been found [163].

### *H. polygyrus*, autoimmunity and allergy

The immunomodulatory properties of *H. polygyrus*, which extend far beyond the site of infection alone, have led to many investigations of the potential for and mechanisms of parasite downregulation of allergic and autoimmune conditions, as discussed below, as well as in the modulation of coinfections with other pathogens (reviewed in [1]).

### Allergy

*H. polygyrus* offers protection in several murine models of allergy, including intestinal, airway, and cutaneous reactions. Mice fed peanut extract administered alongside the mucosal adjuvant cholera toxin produced peanut specific IgE had elevated plasma histamine levels and exhibited systemic anaphylactic shock symptoms. All phenotypes were diminished in *H. polygyrus*-infected mice [164]. In the presence of *H. polygyrus*, the peanut antigen-specific IL-13 levels were drastically reduced, and these dampened IL-13 levels along with protection from peanut allergy were lost when mice were treated with neutralizing IL-10 antibody [164]. The source of IL-10 and the mechanism by which it acts to dampen allergic responses to peanut antigen during *H. polygyrus* infection have not yet been determined.

*H. polygyrus*-infected mice had reduced inflammatory cell infiltrates and bronchoalveolar lavage eosinophilia in experimentally induced airway allergy to both ovalbumin [165–167] and the house dust mite antigen Der p 1 [166]. Protection against these allergens could be transferred by MLN cells from infected mice, which contained a high proportion of CD4^+^CD25^+^Foxp3^+^ T cells, or by transfer of sorted CD4^+^CD25^+^ cells from infected mice, implicating the action of Treg cells in protection [166]. *H. polygyrus-*infected IL-10^−/−^ mice were not protected from ovalbumin-induced asthma [165]; however, MLN cells transferred from IL-10^−/−^ *H. polygyrus* infected mice could still protect from allergy to these antigens, suggesting that IL-10 independent mechanisms can confer protection from allergy [166].

### Inflammatory bowel disease

Inflammatory bowel disease (IBD) is characterized by an inappropriate inflammatory response of the gut to microbial antigens. In humans, there are two main forms of the disease: Crohn’s disease (CD), which can affect the entire length of the gut, and ulcerative colitis (UC), which is localized only to the colon. There are many differing mouse models of IBD (reviewed in [168]), and the effect of *H. polygyrus* infection on controlling the disease has been examined in a number of these models.

IL-10^−/−^ mice suffer from spontaneous chronic colitis [169] associated with excessive IFN-γ production [170]. Spontaneous colitis develops sporadically over several months, but piroxicam treatment will induce rapid and uniform disease in IL-10-deficient mice [171, 172], which likely occurs as a result of increased colonic epithelial cell apoptosis causing a loss of barrier function to inflammatory microbial stimuli [172]. When *H. polygyrus* was given to piroxicam-treated IL-10^−/−^ mice, the histological scores of colitis severity were drastically reduced within 14 days [173, 174]. LPMC from uninfected colitic mice released the inflammatory cytokines IFN-γ, IL-12p40, and IL-17A, whereas LPMC from *H. polygyrus*-infected had significantly reduced levels of these cytokines [173, 174].

Severe colitis also develops when RAG^−/−^ mice are reconstituted with IL-10^−/−^ T cells and treated with piroxicam [175]. *H. polygyrus* colonization reduced gut inflammation in this model, as shown by lower levels of IFN-γ and IL-17 production by restimulated LPMC cells, and a drop in colonic histological score from an average of above 3 (some epithelial and muscle hypertrophy, mucus depletion, crypt abscesses, and epithelial erosions) to less than 1 (some mononuclear cell infiltrates in the lamina propria) [136, 176]. In RAG^−/−^ mice that had been infected with *H. polygyrus*, and subsequently drug-cleared of the infection prior to transfer of the colitogenic IL-10^−/−^ T cells and piroxicam administration, mice still showed reduced levels of inflammation compared to those that had never been infected [176]. The authors reported that protection coincided with downregulation of the costimulatory molecules CD80 and CD86 on DCs, thus inhibiting antigen presentation to T cells resulting in less inflammatory cytokine release [176].

TGF-βRΙΙ DN mice develop spontaneous colitis, which is unable to be suppressed by *H. polygyrus* infection [135]. The inability of *H. polygyrus* to suppress colitis in this model is likely due to the exacerbated IFN-γ levels seen in TGF-βRΙΙ DN mice, which are not dampened during *H. polygyrus* infection [135] and are known to aggravate intestinal inflammation.

Intrarectal injection of trinitrobenzene sulfonic acid (TNBS) administration also induces severe colitis in wild-type mice. When mice that had been infected with *H. polygyrus* for 10 days were given TNBS injection, they exhibited markedly reduced TNBS-induced colonic damage and inflammation and decreased Th1 cytokine mRNA expression compared to uninfected control mice, which was accompanied by increased IL-10 secretion during *H. polygyrus* infection [36, 177].

In contrast to other models of colitis, *H. polygyrus* seems to intensify colitis caused by the murine bacterial pathogen *Citrobacter rodentium* [178–180]. Disease exacerbation could be due to the influx of alternatively activated macrophages during *H. polygyrus* infection, which are less able to kill bacteria than classically activated macrophages [178], or due to increased IL-10 production by DCs impairing mechanisms that kill *C. rodentium*, leading to more persistent infection and colitis [180]. These studies exemplify the need to understand the causes of colitis, and the mechanisms by which helminths modulate disease progression, before helminth therapy can be applied to human inflammatory bowel diseases.

*H. polygyrus* is not the only parasitic nematode shown to have modulatory effects on the onset of colitis; both *T. spiralis* and excretory–secretory products from the hookworms *Ancylostoma caninum* and *Ancylostoma ceylanicum* can also ameliorate colitis progression in murine models, as reviewed in [168]. In human clinical trials, ova from the pig intestinal helminth parasite *Trichuris suis* reduced the severity of disease in some patients with UC and CD [181–183]. Although initial clinical trials with *T. suis* are promising, the fact that the therapy is not effective in 100 % of patients illustrates the need for further studies to understand the immunomodulatory actions of these helminths in murine models of IBD.

### Type 1 diabetes

Nonobese diabetic (NOD) mice spontaneously become diabetic (as measured by blood glucose levels of ≥ 200 mg/dl) by 25 weeks of age [184, 185]. When these mice are infected with *H. polygyrus* at 5 weeks old, the onset of diabetes was completely blocked, at least until 40 weeks of age [184, 185]. Administering *H. polygyrus* when NOD mice were 7 and 12 weeks of age resulted in less effective protection from diabetes, yet onset was still delayed compared to untreated NOD mice [185]. The severity of insulitis (the infiltration of immune cells into the islets of Langerhans) was examined in mice aged 13 weeks, and was sharply reduced in NOD mice infected with *H. polygyrus* since the age of 5 weeks [185]. This reduction was maintained in *H. polygyrus* mice given anti-CD25 antibody [185], suggesting that *H. polygyrus* modulates type 1 diabetes (T1D) onset in a Treg-independent manner, although whether this protection extends beyond the 13-week time point has not been examined.

There is the possibility that the modulatory effects of *H. polygyrus* are due in part to changes in gut microbial composition during infection [186]. Studies to modify the microbial flora could address this, perhaps using fecal transplants, which would allow transfer of the microflora from *H. polygyrus* infected or naive mice to recipient mice using methods described in [187].

## Role of the microbiota

*H. polygyrus* is localized in the anterior small intestine alongside a substantial microbial flora. The presence of specific species of bacteria within the gut is known to polarise naive T cells towards particular Th subset fates [188–190], and as the outcome of *H. polygyrus* infection is dependent on the immediate cytokine environment, it seems reasonable to imagine that commensal microbes may alter the ability of the murine immune system to cause worm expulsion.

Care must therefore be taken when performing experiments to compare the susceptibility to *H. polygyrus* in different mice, as the mice may initially differ in their microbial flora. Variation in microbial flora may be due to the source of mice, as mice of the same strain acquired from different vendors can harbor different gut microbes [188], perhaps due to diet or housing conditions. The genotype of mice can also control microbial populations, as has been shown to be the case for MyD88-deficient mice, which have an altered microbial flora compared to MyD88-sufficient mice as MyD88 controls the release of some antimicrobial peptides [191].

After a 14-day infection with *H. polygyrus* in C57BL/6 mice, the abundance of *Lactobacillaceae* family members was increased in the ileum compared to naive mice [186]. It has yet to be demonstrated whether this shift is a helminth-mediated mechanism that acts to promote the survival of *H. polygyrus* within the murine host, or if it is simply as a consequence of a changing immune environment, in which bacteria of the *Lactobacillaceae* family are better able to survive. To resolve this, further studies to investigate the interplay among parasitic helminths, the microbial flora, and the immune system are necessary.

## Conclusions and implications for human infections and disease

Parasitic helminth infections in humans and livestock are still responsible for unacceptably high levels of morbidity and economic loss worldwide. Understanding the mechanisms necessary for expulsion of the model gastrointestinal parasite *H. polygyrus* is likely to define new pathways, which target the immune system to provide the best protection against other helminth infections. The increasing prevalence of autoimmune diseases in the Western world correlates with the increasing absence of such helminth infections [192]. Our immune systems have evolved to develop in the presence of helminth parasite antigens, and it is vital to understand whether the human immune system can function optimally without this presence. Studies to isolate and understand how immunomodulatory factors secreted by helminths such as *H. polygyrus* act to maintain gut homeostasis are ongoing and will be invaluable both in understanding the interactions between helminths and the immune system and in the development of new pharmaceutical therapies for autoimmune and allergic diseases worldwide.

## References

[1] Maizels RM, Hewitson JP, Murray J, Harcus Y, Dayer B, Filbey KJ, Grainger JR, McSorley HJ, Reynolds LA, Smith KA (2012). Immune modulation and modulators in *Heligmosomoides polygyrus* infection. Exp Parasitol.

[2] Maizels RM, Hewitson JP, Smith KA (2012). Susceptibility and immunity to helminth parasites. Curr Opin Immunol.

[3] Gouy de Bellocq J, Ferte H, Depaquit J, Justine JL, Tillier A, Durette-Desset MC (2001). Phylogeny of the *Trichostrongylina* (Nematoda) inferred from 28S rDNA sequences. Mol Phylogenet Evol.

[4] Bansemir AD, Sukhdeo MVK (1994). The food resource of adult *Heligmosomoides polygyrus* in the small intestine. J Parasitol.

[5] Hewitson JP, Harcus Y, Murray J, van Agtmaal M, Filbey KJ, Grainger JR, Bridgett S, Blaxter ML, Ashton PD, Ashford DA, Curwen RS, Wilson RA, Dowle AA, Maizels RM (2011). Proteomic analysis of secretory products from the model gastrointestinal nematode *Heligmosomoides polygyrus* reveals dominance of Venom Allergen-Like (VAL) proteins. Journal of Proteomics.

[6] Behnke JM, Robinson M (1985). Genetic control of immunity to *Nematospiroides dubius*: a 9-day anthelmintic abbreviated immunizing regime which separates weak and strong responder strains of mice. Parasite Immunol.

[7] Behnke JM, Wahid FN (1991). Immunological relationships during primary infection with *Heligmosomoides polygyrus* (*Nematospiroides dubius*): H-2 linked genes determine worm survival. Parasitology.

[8] Enriquez FJ, Zidian JL, Cypess RH (1988). *Nematospiroides dubius*: genetic control of immunity to infections of mice. Exp Parasitol.

[9] Wahid FN, Behnke JM (1993). Immunological relationships during primary infection with *Heligmosomoides polygyrus*. Regulation of fast response phenotype by H-2 and non-H-2 genes. Parasitology.

[10] Behnke JM, Iraqi F, Menge D, Baker RL, Gibson J, Wakelin D (2003). Chasing the genes that control resistance to gastrointestinal nematodes. J Helminthol.

[11] Dobson C, Owen ME (1978). Effect of host sex on passive immunity in mice infected with *Nematospiroides dubius*. Int J Parasitol.

[12] Wahid FN, Behnke JM (1993). Immunological relationships during primary infection with *Heligmosomoides polygyrus* (*Nematospiroides dubius*): parasite specific IgG1 antibody responses and primary response phenotype. Parasite Immunol.

[13] Van Zandt PD, Cypess RH, Zidian JL (1973). Development of age and sex resistance to *Nematospiroides dubius* in the mouse following single and multiple infections. J Parasitol.

[14] Zhong S, Dobson C (1996). *Heligmosomoides polygyrus:* resistance in inbred, outbred, and selected mice. Exp Parasitol.

[15] Odiere MR, Koski KG, Weiler HA, Scott ME (2010). Concurrent nematode infection and pregnancy induce physiological responses that impair linear growth in the murine foetus. Parasitology.

[16] Kristan DM (2002). Effects of intestinal nematodes during lactation: consequences for host morphology, physiology and offspring mass. J Exp Biol.

[17] Crump A, Omura S (2011). Ivermectin, ‘wonder drug’ from Japan: the human use perspective. Proc Jpn Acad Ser B Phys Biol Sci.

[18] Finkelman FD, Shea-Donohue T, Goldhill J, Sullivan CA, Morris SC, Madden KB, Gause WC, Urban JF (1997). Cytokine regulation of host defense against parasitic gastrointestinal nematodes: lessons from studies with rodent models. Annu Rev Immunol.

[19] Scott ME (1991). *Heligmosomoides polygyrus* (Nematoda): susceptible and resistant strains of mice are indistinguishable following natural infection. Parasitology.

[20] Prowse SJ, Mitchell GF, Ley PL, Jenkin CR (1979). The development of resistance in different inbred strains of mice to infection with *Nematospiroides dubius*. Parasite Immunology.

[21] Hewitson JP, Filbey KJ, Grainger JR, Dowle AA, Pearson M, Murray J, Harcus Y, Maizels RM (2011). *Heligmosomoides polygyrus* elicits a dominant nonprotective antibody response directed at restricted glycan and peptide epitopes. J Immunol.

[22] Brailsford TJ, Behnke JM (1992). The dynamics of trickle infections with *Heligmosomoides polygyrus* in syngeneic strains of mice. Int J Parasitol.

[23] Behnke J, Harris PD (2010). *Heligmosomoides bakeri:* a new name for an old worm?. Trends Parasitol.

[24] Behnke JM, Keymer AE, Lewis JW (1991). *Heligmosomoides polygyrus* or *Nematospiroides dubius* ?. Parasitology Today.

[25] Cable J, Harris PD, Lewis JW, Behnke JM (2006). Molecular evidence that *Heligmosomoides polygyrus* from laboratory mice and wood mice are separate species. Parasitology.

[26] Maizels RM, Hewitson JP, Gause WC (2011). *Heligmosomoides polygyrus*: one species still. Trends Parasitol.

[27] Tang J, Dobson C, McManus DP (1995). Antigens in phenotypes of *Heligmosomoides polygyrus* raised selectively from different strains of mice. Int J Parasitol.

[28] Morgan C, LaCourse EJ, Rushbrook BJ, Greetham D, Hamilton JV, Barrett J, Bailey K, Brophy PM (2006). Plasticity demonstrated in the proteome of a parasitic nematode within the intestine of different host strains. Proteomics.

[29] Urban JF, Maliszewski CR, Madden KB, Katona IM, Finkelman FD (1995). IL-4 treatment can cure established gastrointestinal nematode infections in immunocompetent and immunodeficient mice. J Immunol.

[30] Hashimoto K, Uchikawa R, Tegoshi T, Takeda K, Yamada M, Arizono N (2009). Depleted intestinal goblet cells and severe pathological changes in SCID mice infected with *Heligmosomoides polygyrus*. Parasite Immunol.

[31] Urban JF, Katona IM, Finkelman FD (1991). *Heligmosomoides polygyrus:* CD4+ but not CD8+ T cells regulate the IgE response and protective immunity in mice. Exp Parasitol.

[32] Rausch S, Huehn J, Kirchhoff D, Rzepecka J, Schnoeller C, Pillai S, Loddenkemper C, Scheffold A, Hamann A, Lucius R, Hartmann S (2008). Functional analysis of effector and regulatory T cells in a parasitic nematode infection. Infect Immun.

[33] Harris N, Gause WC (2011). To B or not to B: B cells and the Th2-type immune response to helminths. Trends Immunol.

[34] Svetić A, Madden KB, di Zhou X, Lu P, Katona IM, Finkelman FD, Urban JF, Gause WC (1993). A primary intestinal helminthic infection rapidly induces a gut-associated elevation of Th2-associated cytokines and IL-3. J Immunol.

[35] Finney CAM, Taylor MD, Wilson MS, Maizels RM (2007). Expansion and activation of CD4^+^CD25^+^ regulatory T cells in *Heligmosomoides polygyrus* infection. Eur J Immunol.

[36] Setiawan T, Metwali A, Blum AM, Ince MN, Urban JF, Elliott DE, Weinstock JV (2007). *Heligmosomoides polygyrus* promotes regulatory T-cell cytokine production in the murine normal distal intestine. Infect Immun.

[37] Mohrs K, Harris DP, Lund FE, Mohrs M (2005). Systemic dissemination and persistence of Th2 and type 2 cells in response to infection with a strictly enteric nematode parasite. J Immunol.

[38] Doligalska M, Donskow-Schmelter K, Rzepecka J, Drela N (2007). Reduced apoptosis in BALB/c mice infected with *Heligmosomoides polygyrus*. Parasite Immunol.

[39] Lu P, di Zhou X, Chen S-J, Moorman M, Morris SC, Finkelman FD, Lionsley P, Urban JF, Gause WC (1994). CTLA-4 ligands are required to induce an in vivo interleukin 4 response to a gastrointestinal nematode parasite. Journal of Experimental Medicine.

[40] Greenwald RJ, Lu P, Halvorson MJ, Zhou X, Chen S, Madden KB, Perrin PJ, Morris SC, Finkelman FD, Peach R, Linsley PS, Urban JF, Gause WC (1997). Effects of blocking B7-1 and B7-2 interactions during a type 2 in vivo immune response. J Immunol.

[41] Greenwald RJ, Urban JF, Ekkens MJ, Chen S, Nguyen D, Fang H, Finkelman FD, Sharpe AH, Gause WC (1999). B7-2 is required for the progression but not the initiation of the type 2 immune response to a gastrointestinal nematode parasite. J Immunol.

[42] Gause WC, Chen SJ, Greenwald RJ, Halvorson MJ, Lu P, Zhou XD, Morris SC, Lee KP, June CH, Finkelman FD, Urban JF, Abe R (1997). CD28 dependence of T cell differentiation to IL-4 production varies with the particular type 2 immune response. J Immunol.

[43] Gause WC, Lu P, Di Zhou X, Chen S-J, Madden KB, Morris SC, Linsley PS, Finkelman FD, Urban JF (1996). *H.polygyrus* : B7-independence of the secondary type 2 response. Exp Parasitol.

[44] Ekkens MJ, Liu Z, Liu Q, Foster A, Whitmire J, Pesce J, Sharpe AH, Urban JF, Gause WC (2002). Memory Th2 effector cells can develop in the absence of B7-1/B7-2, CD28 interactions, and effector Th cells after priming with an intestinal nematode parasite. J Immunol.

[45] Ekkens MJ, Liu Z, Liu Q, Whitmire J, Xiao S, Foster A, Pesce J, VanNoy J, Sharpe AH, Urban JF, Gause WC (2003). The role of OX40 ligand interactions in the development of the Th2 response to the gastrointestinal nematode parasite *Heligmosomoides polygyrus*. J Immunol.

[46] King C, Tangye SG, Mackay CR (2008). T follicular helper (TFH) cells in normal and dysregulated immune responses. Annu Rev Immunol.

[47] King IL, Mohrs M (2009). IL-4-producing CD4+ T cells in reactive lymph nodes during helminth infection are T follicular helper cells. J Exp Med.

[48] Yi JS, Cox MA, Zajac AJ (2010). Interleukin-21: a multifunctional regulator of immunity to infections. Microbes Infect.

[49] Fröhlich A, Marsland BJ, Sonderegger I, Kurrer M, Hodge MR, Harris NL, Kopf M (2007). IL-21 receptor signaling is integral to the development of Th2 effector responses in vivo. Blood.

[50] King IL, Mohrs K, Mohrs M (2010). A nonredundant role for IL-21 receptor signaling in plasma cell differentiation and protective type 2 immunity against gastrointestinal helminth infection. J Immunol.

[51] Ben-Smith A, Lammas DA, Behnke JM (2003). The relative involvement of Th1 and Th2 associated immune responses in the expulsion of a primary infection of *Heligmosomoides polygyrus* in mice of differing response phenotype. J Helminthol.

[52] Wahid FN, Behnke JM, Grencis RK, Else KJ, Ben-Smith AW (1994). Immunological relationships during primary infection with *Heligmosomoides polygyrus:* Th2 cytokines and primary response phenotype. Parasitology.

[53] Behnke JM, Lowe A, Clifford S, Wakelin D (2003). Cellular and serological responses in resistant and susceptible mice exposed to repeated infection with *Heligmosomoides polygyrus bakeri*. Parasite Immunol.

[54] Alugupalli KR, Abraham D (2009). B cell multitasking is required to control nematode infection. Immunity.

[55] Harris DP, Haynes L, Sayles PC, Duso DK, Eaton SM, Lepak NM, Johnson LL, Swain SL, Lund FE (2000). Reciprocal regulation of polarized cytokine production by effector B and T cells. Nat Immunol.

[56] Wilson MS, Taylor MD, O’Gorman MT, Balic A, Barr TA, Filbey K, Anderton SM, Maizels RM (2010). Helminth-induced CD19^+^CD23^hi^ B cells modulate experimental allergic and autoimmune inflammation. Eur J Immunol.

[57] Wojciechowski W, Harris DP, Sprague F, Mousseau B, Makris M, Kusser K, Honjo T, Mohrs K, Mohrs M, Randall T, Lund FE (2009). Cytokine-producing effector B cells regulate type 2 immunity to *H. polygyrus*. Immunity.

[58] Liu Q, Kreider T, Bowdridge S, Liu Z, Song Y, Gaydo AG, Urban JF, Gause WC (2010). B cells have distinct roles in host protection against different nematode parasites. J Immunol.

[59] McCoy KD, Stoel M, Stettler R, Merky P, Fink K, Senn BM, Schaer C, Massacand J, Odermatt B, Oettgen HC, Zinkernagel RM, Bos NA, Hengartner H, Macpherson AJ, Harris NL (2008). Polyclonal and specific antibodies mediate protective immunity against enteric helminth infection. Cell Host Microbe.

[60] Blackwell NM, Else KJ (2001). B cells and antibodies are required for resistance to the parasitic gastrointestinal nematode *Trichuris muris*. Infect Immun.

[61] Pritchard DI, Williams DJL, Behnke JM, Lee TDG (1983). The role of IgG1 hypergammaglobulinaemia in immunity to the gastrointestinal nematode *Nematospiroides dubius.* The immunochemical purification, antigen-specificity and in vivo anti-parasite effect of IgG1 from immune serum. Immunology.

[62] Prowse SJ, Ey PL, Jenkin CR (1978). Immunity to *Nematospiroides dubius*: cell and immunoglobulin changes associated with the onset of immunity in mice. Aust J Exp Biol Med Sci.

[63] Chapman CB, Knopf PM, Hicks JD, Mitchell GF (1979). IgG1 hypergammaglobulinaemia in chronic parasitic infections in mice: magnitude of the response in mice infected with various parasites. Aust J Exp Biol Med Sci.

[64] Williams DJ, Behnke JM (1983). Host protective antibodies and serum immunoglobulin isotypes in mice chronically infected or repeatedly immunized with the nematode parasite *Nematospiroides dubius*. Immunology.

[65] Harris NL, Spoerri I, Schopfer JF, Nembrini C, Merky P, Massacand J, Urban JF, Lamarre A, Burki K, Odermatt B, Zinkernagel RM, Macpherson AJ (2006). Mechanisms of neonatal mucosal antibody protection. J Immunol.

[66] Allen JE, Maizels RM (2011). Diversity and dialogue in immunity to helminths. Nat Rev Immunol.

[67] Neill DR, McKenzie ANJ (2011). Nuocytes and beyond: new insights into helminth expulsion. Trends Parasitol.

[68] MacDonald AS, Maizels RM (2008). Alarming dendritic cells for Th2 induction. J Exp Med.

[69] Balic A, Harcus Y, Holland MJ, Maizels RM (2004). Selective maturation of dendritic cells by *Nippostrongylus brasiliensis* secreted proteins drives T helper type 2 immune responses. Eur J Immunol.

[70] Liu JY, Li LY, Yang XZ, Li J, Zhong G, Wang J, Li LJ, Ji B, Wu ZQ, Liu H, Yang X, Liu PM (2011) Adoptive transfer of DCs isolated from helminth-infected mice enhanced T regulatory cell responses in airway allergic inflammation. Parasite Immunol 33:525–534 10.1111/j.1365-3024.2011.01308.x 21711363

[71] Hochweller K, Striegler J, Hammerling GJ, Garbi N (2008). A novel CD11c.DTR transgenic mouse for depletion of dendritic cells reveals their requirement for homeostatic proliferation of natural killer cells. Eur J Immunol.

[72] Phythian-Adams AT, Cook PC, Lundie RJ, Jones LH, Smith KA, Barr TA, Hochweller K, Anderton SM, Hämmerling GJ, Maizels RM, MacDonald AS (2010). CD11c depletion severely disrupts Th2 induction and development in vivo. J Exp Med.

[73] Ohnmacht C, Pullner A, King SB, Drexler I, Meier S, Brocker T, Voehringer D (2009). Constitutive ablation of dendritic cells breaks self-tolerance of CD4 T cells and results in spontaneous fatal autoimmunity. J Exp Med.

[74] Anthony RM, Rutitzky LI, Urban JF, Stadecker MJ, Gause WC (2007). Protective immune mechanisms in helminth infection. Nat Rev Immunol.

[75] Modolell M, Corraliza IM, Link F, Soler G, Eichmann K (1995). Reciprocal regulation of the nitric oxide synthase/arginase balance in mouse bone marrow-derived macrophages by TH1 and TH2 cytokines. Eur J Immunol.

[76] Hesse M, Modolell M, La Flamme AC, Schito M, Fuentes JM, Cheever AW, Pearce EJ, Wynn TA (2001). Differential regulation of nitric oxide synthase-2 and arginase-1 by type 1/type 2 cytokines in vivo: Granulomatous pathology is shaped by the pattern of L-arginine metabolism. J Immunol.

[77] Pesce J, Kaviratne M, Ramalingam TR, Thompson RW, Urban JF, Cheever AW, Young DA, Collins M, Grusby MJ, Wynn TA (2006). The IL-21 receptor augments Th2 effector function and alternative macrophage activation. J Clin Invest.

[78] Park-Min KH, Antoniv TT, Ivashkiv LB (2005). Regulation of macrophage phenotype by long-term exposure to IL-10. Immunobiology.

[79] Mylonas KJ, Nair MG, Prieto-Lafuente L, Paape D, Allen JE (2009). Alternatively activated macrophages elicited by helminth infection can be reprogrammed to enable microbial killing. J Immunol.

[80] Anthony RM, Urban JF, Alem F, Hamed HA, Rozo CT, Boucher JL, Van Rooijen N, Gause WC (2006). Memory T_H_2 cells induce alternatively activated macrophages to mediate protection against nematode parasites. Nat Med.

[81] Chang N-CA, Hung S-I, Hwa K-Y, Kato I, Chen J-E, Liu C-H, Chang AC (2001). A macrophage protein, Ym1, transiently expressed during inflammation is a novel mammalian lectin. J Biol Chem.

[82] Loke P, Gallagher I, Nair MG, Zang X, Brombacher F, Mohrs M, Allison JP, Allen JE (2007). Alternative activation is an innate response to injury that requires CD4^+^ T cells to be sustained during chronic infection. J Immunol.

[83] Zhao A, Urban JF, Anthony RM, Sun R, Stiltz J, van Rooijen N, Wynn TA, Gause WC, Shea-Donohue T (2008). Th2 cytokine-induced alterations in intestinal smooth muscle function depend on alternatively activated macrophages. Gastroenterology.

[84] Morimoto M, Morimoto M, Whitmire J, Xiao S, Anthony RM, Mirakami H, Star RA, Urban JF, Gause WC (2004). Peripheral CD4 T cells rapidly accumulate at the host: parasite interface during an inflammatory Th2 memory response. J Immunol.

[85] Patel N, Kreider T, Urban JF, Gause WC (2009). Characterisation of effector mechanisms at the host: parasite interface during the immune response to tissue-dwelling intestinal nematode parasites. Int J Parasitol.

[86] Galioto AM, Hess JA, Nolan TJ, Schad GA, Lee JJ, Abraham D (2006). Role of eosinophils and neutrophils in innate and adaptive protective immunity to larval strongyloides stercoralis in mice. Infect Immun.

[87] O’Connell AE, Hess JA, Santiago GA, Nolan TJ, Lok JB, Lee JJ, Abraham D (2011). Major basic protein from eosinophils and myeloperoxidase from neutrophils are required for protective immunity to *Strongyloides stercoralis* in mice. Infect Immun.

[88] Yu C, Cantor AB, Yang H, Browne C, Wells RA, Fujiwara Y, Orkin SH (2002). Targeted deletion of a high-affinity GATA-binding site in the GATA-1 promoter leads to selective loss of the eosinophil lineage in vivo. J Exp Med.

[89] Knott ML, Matthaei KI, Giacomin PR, Wang H, Foster PS, Dent LA (2007). Impaired resistance in early secondary *Nippostrongylus brasiliensis* infections in mice with defective eosinophilopoeisis. Int J Parasitol.

[90] Khan WI, Abe T, Ishikawa N, Nawa Y, Yoshimura K (1995). Reduced amount of intestinal mucus by treatment with anti-CD4 antibody interferes with the spontaneous cure of *Nippostrongylus brasiliensis*-infection in mice. Parasite Immunology.

[91] Rennick DM, Thompson-Snipes L, Coffman RL, Seymour BW, Jackson JD, Hudak S (1990). In vivo administration of antibody to interleukin-5 inhibits increased generation of eosinophils and their progenitors in bone marrow of parasitized mice. Blood.

[92] Urban JF, Katona IM, Paul WE, Finkelman FD (1991). Interleukin 4 is important in protective immunity to a gastrointestinal nematode infection in mice. Proceedings of the National Academy of Sciences USA.

[93] Doligalska M, Rzepecka J, Drela N, Donskow K, Gerwel-Wronka M (2006). The role of TGF-β in mice infected with *Heligmosomoides polygyrus*. Parasite Immunol.

[94] Ohnmacht C, Voehringer D (2009). Basophil effector function and homeostasis during helminth infection. Blood.

[95] Perrigoue JG, Saenz SA, Siracusa MC, Allenspach EJ, Taylor BC, Giacomin PR, Nair MG, Du Y, Zaph C, van Rooijen N, Comeau MR, Pearce EJ, Laufer TM, Artis D (2009). MHC class II-dependent basophil-CD4^+^ T cell interactions promote TH2 cytokine-dependent immunity. Nat Immunol.

[96] Behnke JM, Wahid FN, Grencis RK, Else KJ, Ben-Smith AW, Goyal PK (1993). Immunological relationships during primary infection with *Heligmosomoides polygyrus* (*Nematospiroides dubius*): downregulation of specific cytokine secretion (IL-9 and IL-10) correlates with poor mastocytosis and chronic survival of adult worms. Parasite Immunology.

[97] Snoek SA, Dhawan S, van Bree SH, Cailotto C, van Diest SA, Duarte JM, Stanisor OI, Hilbers FW, Nijhuis L, Koeman A, van den Wijngaard RM, Zuurbier CJ, Boeckxstaens GE, de Jonge WJ (2012) Mast cells trigger epithelial barrier dysfunction, bacterial translocation and postoperative ileus in a mouse model. Neurogastroenterol Motil 24:172–191 10.1111/j.1365-2982.2011.01820.x 22122661

[98] McDermott JR, Bartram RE, Knight PA, Miller HR, Garrod DR, Grencis RK (2003). Mast cells disrupt epithelial barrier function during enteric nematode infection. Proc Natl Acad Sci U S A.

[99] Dehlawi MS, Wakelin D, Behnke JM (1987). Suppression of mucosal mastocytosis by infection with the intestinal nematode *Nematospiroides dubius*. Parasite Immunology.

[100] Hashimoto K, Uchikawa R, Tegoshi T, Takeda K, Yamada M, Arizono N (2010). Immunity-mediated regulation of fecundity in the nematode *Heligmosomoides polygyrus*—the potential role of mast cells. Parasitology.

[101] Morimoto M, Utsumiya K (2011). Enhanced protection against *Heligmosomoides polygyrus* in IL-2 receptor β-chain overexpressed transgenic mice with intestinal mastocytosis. J Vet Med Sci.

[102] Hepworth MR, Danilowicz-Luebert E, Rausch S, Metz M, Klotz C, Maurer M, Hartmann S (2012). Mast cells orchestrate type 2 immunity to helminths through regulation of tissue-derived cytokines. Proc Natl Acad Sci U S A.

[103] Grimbaldeston MA, Chen CC, Piliponsky AM, Tsai M, Tam SY, Galli SJ (2005). Mast cell-deficient W-sash c-kit mutant Kit W-sh/W-sh mice as a model for investigating mast cell biology in vivo. Am J Pathol.

[104] Barlow JL, McKenzie AN (2011) Nuocytes: expanding the innate cell repertoire in type-2 immunity. J Leukoc Biol 90:867–874 10.1189/jlb.0311160 21712394

[105] Ikeda K, Nakajima H, Suzuki K, Kagami S, Hirose K, Suto A, Saito Y, Iwamoto I (2003). Mast cells produce interleukin-25 upon Fc epsilon RI-mediated activation. Blood.

[106] Paul WE, Zhu J (2010). How are T_H_2-type immune responses initiated and amplified?. Nat Rev Immunol.

[107] Wills-Karp M, Rani R, Dienger K, Lewkowich I, Fox JG, Perkins C, Lewis L, Finkelman FD, Smith DE, Bryce PJ, Kurt-Jones EA, Wang TC, Sivaprasad U, Hershey GK, Herbert DR (2012). Trefoil factor 2 rapidly induces interleukin 33 to promote type 2 immunity during allergic asthma and hookworm infection. J Exp Med.

[108] Fallon PG, Ballantyne SJ, Mangan NE, Barlow JL, Dasvarma A, Hewett DR, McIlgorm A, Jolin HE, McKenzie ANJ (2006). Identification of an interleukin (IL)-25-dependent cell population that provides IL-4, IL-5, and IL-13 at the onset of helminth expulsion. J Exp Med.

[109] Neill DR, Wong SH, Bellosi A, Flynn RJ, Daly M, Langford TKA, Bucks C, Kane CM, Fallon PG, Pannell R, Jolin HE, McKenzie ANJ (2010). Nuocytes represent a new innate effector leukocyte that mediates type-2 immunity. Nature.

[110] Saenz SA, Siracusa MC, Perrigoue JG, Spencer SP, Urban JF, Tocker JE, Budelsky AL, Kleinschek MA, Kastelein RA, Kambayashi T, Bhandoola A, Artis D (2010). IL25 elicits a multipotent progenitor cell population that promotes T_H_2 cytokine responses. Nature.

[111] Shea-Donohue T, Sullivan C, Finkelman FD, Madden KB, Morris SC, Goldhill J, Pineiro-Carrero V, Urban JF (2001). The role of IL-4 in *Heligmosomoides polygyrus*-induced alterations in murine intestinal epithelial cell function. J Immunol.

[112] Madden KB, Yeung KA, Zhao A, Gause WC, Finkelman FD, Katona IM, Urban JF, Shea-Donohue T (2004). Enteric nematodes induce stereotypic STAT6-dependent alterations in intestinal epithelial cell function. J Immunol.

[113] Artis D, Wang ML, Keilbaugh SA, He W, Brenes M, Swain GP, Knight PA, Donaldson DD, Lazar MA, Miller HR, Schad GA, Scott P, Wu GD (2004). RELMβ/FIZZ2 is a goblet cell-specific immune-effector molecule in the gastrointestinal tract. Proceedings of the National Academy of Sciences USA.

[114] Hogan SP, Seidu L, Blanchard C, Groschwitz K, Mishra A, Karow ML, Ahrens R, Artis D, Murphy AJ, Valenzuela DM, Yancopoulos GD, Rothenberg ME (2006). Resistin-like molecule beta regulates innate colonic function: Barrier integrity and inflammation susceptibility. J Allergy Clin Immunol.

[115] Herbert DR, Yang J-Q, Hogan SP, Groschwitz K, Khodoun MV, Munitz A, Orekov T, Perkins C, Wang Q, Brombacher F, Urban JF, Rothenberg ME, Finkelman FD (2009). Intestinal epithelial cell secretion of RELM-β protects against gastrointestinal worm infection. J Exp Med.

[116] Hasnain SZ, Wang H, Ghia JE, Haq N, Deng Y, Velcich A, Grencis RK, Thornton DJ, Khan WI (2010). Mucin gene deficiency in mice impairs host resistance to an enteric parasitic infection. Gastroenterology.

[117] Hasnain SZ, Evans CM, Roy M, Gallagher AL, Kindrachuk KN, Barron L, Dickey BF, Wilson MS, Wynn TA, Grencis RK, Thornton DJ (2011). Muc5ac: a critical component mediating the rejection of enteric nematodes. J Exp Med.

[118] Inagaki-Ohara K, Sakamoto Y, Dohi T, Smith AL (2011). gammadelta T cells play a protective role during infection with *Nippostrongylus brasiliensis* by promoting goblet cell function in the small intestine. Immunology.

[119] Zhao A, McDermott J, Urban JF, Gause W, Madden KB, Yeung KA, Morris SC, Finkelman FD, Shea-Donohue T (2003). Dependence of IL-4, IL-13, and nematode-induced alterations in murine small intestinal smooth muscle contractility on Stat6 and enteric nerves. J Immunol.

[120] Marillier RG, Brombacher TM, Dewals B, Leeto M, Barkhuizen M, Govender D, Kellaway L, Horsnell WG, Brombacher F (2010). IL-4Rα-responsive smooth muscle cells increase intestinal hypercontractility and contribute to resistance during acute Schistosomiasis. Am J Physiol Gastrointest Liver Physiol.

[121] Vallance BA, Blennerhassett PA, Collins SM (1997). Increased intestinal muscle contractility and worm expulsion in nematode-infected mice. Am J Physiol.

[122] Vallance BA, Blennerhassett PA, Deng Y, Matthaei KI, Young IG, Collins SM (1999). IL-5 contributes to worm expulsion and muscle hypercontractility in a primary *T. spiralis* infection. Am J Physiol.

[123] Zhao M, Brown DM, Maccallum J, Proudfoot L (2009). Effect of *Nippostrongylus brasiliensis* L3 ES on inflammatory mediator gene transcription in lipopolysaccharide lung inflammation. Parasite Immunol.

[124] Shea-Donohue T, Notari L, Stiltz J, Sun R, Madden KB, Urban JF, Zhao A (2010). Role of enteric nerves in immune-mediated changes in protease-activated receptor 2 effects on gut function. Neurogastroenterol Motil.

[125] Menge DM, Behnke JM, Lowe A, Gibson JP, Iraqi FA, Baker RL, Wakelin D (2003). Mapping of chromosomal regions influencing immunological responses to gastrointestinal nematode infections in mice. Parasite Immunol.

[126] Martin P, Leibovich SJ (2005). Inflammatory cells during wound repair: the good, the bad and the ugly. Trends Cell Biol.

[127] Williams LM, Rudensky AY (2007). Maintenance of the Foxp3-dependent developmental program in mature regulatory T cells requires continued expression of Foxp3. Nat Immunol.

[128] Fontenot JD, Gavin MA, Rudensky AY (2003). Foxp3 programs the development and function of CD4^+^CD25^+^ regulatory T cells. Nat Immunol.

[129] Belkaid Y, Tarbell K (2009). Regulatory T cells in the control of host-microorganism interactions. Annu Rev Immunol.

[130] Rausch S, Huehn J, Loddenkemper C, Hepworth MR, Klotz C, Sparwasser T, Hamann A, Lucius R, Hartmann S (2009). Establishment of nematode infection despite increased Th2 responses and immunopathology after selective depletion of Foxp3^+^ cells. Eur J Immunol.

[131] Murai M, Krause P, Cheroutre H, Kronenberg M (2010). Regulatory T-cell stability and plasticity in mucosal and systemic immune systems. Mucosal Immunol.

[132] Grainger JR, Smith KA, Hewitson JP, McSorley HJ, Harcus Y, Filbey KJ, Finney CAM, Greenwood EJD, Knox DP, Wilson MS, Belkaid Y, Rudensky AY, Maizels RM (2010). Helminth secretions induce *de novo* T cell Foxp3 expression and regulatory function through the TGF-β pathway. J Exp Med.

[133] Gorelik L, Flavell RA (2000). Abrogation of TGFβ signaling in T cells leads to spontaneous T cell differentiation and autoimmune disease. Immunity.

[134] Reynolds LA, Maizels RM (2012). Cutting edge: in the absence of TGF-β signaling in T cells, fewer CD103+ regulatory T cells develop, but exuberant IFN-gamma production renders mice more susceptible to helminth infection. J Immunol.

[135] Ince MN, Elliott DE, Setiawan T, Metwali A, Blum A, Chen HL, Urban JF, Flavell RA, Weinstock JV (2009). Role of T cell TGF-β signaling in intestinal cytokine responses and helminthic immune modulation. Eur J Immunol.

[136] Metwali A, Setiawan T, Blum AM, Urban J, Elliott DE, Hang L, Weinstock JV (2006). Induction of CD8^+^ regulatory T cells in the intestine by *Heligmosomoides polygyrus* infection. Am J Physiol Gastrointest Liver Physiol.

[137] Mizoguchi A, Bhan AK (2006). A case for regulatory B cells. J Immunol.

[138] Jankovic D, Cheever AW, Kullberg MC, Wynn TA, Yap G, Caspar P, Lewis FA, Clynes R, Ravetch JV, Sher A (1998). CD4^+^ T cell-mediated granulomatous pathology in schistosomiasis is downregulated by B cell-dependent mechanism requiring Fc receptor signaling. Journal of Experimental Medicine.

[139] Correale J, Farez M, Razzitte G (2008). Helminth infections associated with multiple sclerosis induce regulatory B cells. Ann Neurol.

[140] Mangan NE, Fallon RE, Smith P, van Rooijen N, McKenzie AN, Fallon PG (2004). Helminth infection protects mice from anaphylaxis via IL-10-producing B cells. J Immunol.

[141] Balic A, Smith KA, Harcus Y, Maizels RM (2009). Dynamics of CD11c^+^ dendritic cell subsets in lymph nodes draining the site of intestinal nematode infection. Immunol Lett.

[142] Smith KA, Hochweller K, Hämmerling GJ, Boon L, Macdonald AS, Maizels RM (2011). Chronic helminth infection mediates tolerance in vivo through dominance of CD11c^lo^ CD103^–^ DC population. J Immunol.

[143] Li Z, Liu G, Chen Y, Liu Y, Liu B, Su Z (2011). The phenotype and function of naturally existing regulatory dendritic cells in nematode-infected mice. Int J Parasitol.

[144] Segura M, Su Z, Piccirillo C, Stevenson MM (2007). Impairment of dendritic cell function by excretory–secretory products: a potential mechanism for nematode-induced immunosuppression. Eur J Immunol.

[145] Chaicumpa V, Prowse SJ, Ey PL, Jenkin CR (1977). Induction of immunity in mice to the nematode parasite, *Nematospiroides dubius*. Australian Journal of Experimental Biology and Medicine.

[146] Behnke JM, Hannah J, Pritchard DI (1983). *Nematospiroides dubius* in the mouse: evidence that adult worms depress the expression of homologous immunity. Parasite Immunology.

[147] Pleass RJ, Bianco AE (1996). Irradiated larval vaccination and antibody responses evaluated in relation to the expression of immunity to *Heligmosomoides polygyrus*. Parasitol Res.

[148] Hagan P, Behnke JM, Parish HA (1981). Stimulation of immunity to *Nematospiroides dubius* in mice using larvae attentuated by cobalt 60 irradiation. Parasite Immunology.

[149] Pleass RJ, Bianco AE (1994). The role of adult worms in suppressing functional protective immunity to *Heligmosomoides polygyrus bakeri* challenge infections. Parasite Immunology.

[150] Larrick KS, Semprevivo LH, Maloney MD, Tritschler JP (1991). Immunity to *Heligmosomoides polygyrus* induced by subcutaneous vaccination with post-infection larvae. Int J Parasitol.

[151] Monroy FG, East IJ, Dobson C, Adams JH (1989). Immunity in mice vaccinated with a molecular weight 60,000 glycoprotein secreted by adult *Nematospiroides dubius*. Int J Parasitol.

[152] Hewitson JP, Grainger JR, Maizels RM (2009). Helminth immunoregulation: the role of parasite secreted proteins in modulating host immunity. Mol Biochem Parasitol.

[153] Harnett W, Harnett MM (2010). Helminth-derived immunomodulators: can understanding the worm produce the pill?. Nat Rev Immunol.

[154] Monroy FG, Dobson C, Adams JH (1989). Low molecular weight immunosuppressors secreted by adult *Nematospiroides dubius*. Int J Parasitol.

[155] Telford G, Wheeler DJ, Appleby P, Bowen JG, Pritchard DI (1998). *Heligmosomoides polygyrus* immunomodulatory factor (IMF), targets T- lymphocytes. Parasite Immunology.

[156] Massacand JC, Stettler RC, Meier R, Humphreys NE, Grencis RK, Marsland BJ, Harris NL (2009). Helminth products bypass the need for TSLP in Th2 immune responses by directly modulating dendritic cell function. Proc Natl Acad Sci U S A.

[157] McSorley HJ, O’Gorman MT, Blair N, Sutherland TE, Filbey KJ, Maizels RM (2012) Suppression of type 2 immunity and allergic airway inflammation by secreted products of the helminth. Heligmosomoides polygyrus. Eur J Immunol. doi:10.1002/eji.201142161 10.1002/eji.201142161 PMC4916998 22706967

[158] Moreno Y, Gros PP, Tam M, Segura M, Valanparambil R, Geary TG, Stevenson MM (2011). Proteomic analysis of excretory-secretory products of *Heligmosomoides polygyrus* assessed with next-generation sequencing transcriptomic information. PLoS Negl Trop Dis.

[159] Lawrence CE, Pritchard DI (1993). Differential secretion of acetylcholinesterase and proteases during the development of *Heligmosomoides polygyrus*. Int J Parasitol.

[160] Ey PL (1988). *Heligmosomoides polygyrus*: excretory/secretory antigens released in vitro by exsheathed third-stage larvae. Exp Parasitol.

[161] Rzepecka J, Rausch S, Klotz C, Schnöller C, Kornprobst T, Hagen J, Ignatius R, Lucius R, Hartmann S (2009). Calreticulin from the intestinal nematode *Heligmosomoides polygyrus* is a Th2-skewing protein and interacts with murine scavenger receptor-A. Mol Immunol.

[162] McSorley HJ, Grainger JR, Harcus Y, Murray J, Nisbet AJ, Knox DP, Maizels RM (2010). daf-7-related TGF-β homologues from Trichostrongyloid nematodes show contrasting life-cycle expression patterns. Parasitology.

[163] Adams JH, East IJ, Monroy FG, Dobson C (1988). Sex-specific antigens on the surface and in the secretions of *Nematospiroides dubius*. Int J Parasitol.

[164] Bashir ME, Andersen P, Fuss IJ, Shi HN, Nagler-Anderson C (2002). An enteric helminth infection protects against an allergic response to dietary antigen. J Immunol.

[165] Kitagaki K, Businga TR, Racila D, Elliott DE, Weinstock JV, Kline JN (2006). Intestinal helminths protect in a murine model of asthma. J Immunol.

[166] Wilson MS, Taylor M, Balic A, Finney CAM, Lamb JR, Maizels RM (2005). Suppression of allergic airway inflammation by helminth-induced regulatory T cells. Journal of Experimental Medicine.

[167] Hartmann S, Schnoeller C, Dahten A, Avagyan A, Rausch S, Lendner M, Bocian C, Pillai S, Loddenkemper C, Lucius R, Worm M, Hamelmann E (2009). Gastrointestinal nematode infection interferes with experimental allergic airway inflammation but not atopic dermatitis. Clin Exp Allergy.

[168] Whelan RAK, Hartmann S, Rausch S (2011) Nematode modulation of inflammatory bowel disease. Protoplasma doi:10.1007/s00709-011-0342-x 10.1007/s00709-011-0342-x PMC3459088 22086188

[169] Kühn R, Löhler J, Rennick D, Rajewsky K, Müller W (1993). Interleukin-10-deficient mice develop chronic enterocolitis. Cell.

[170] Berg DJ, Davidson N, Kuhn R, Muller W, Menon S, Holland G, Thompson-Snipes L, Leach MW, Rennick D (1996). Enterocolitis and colon cancer in interleukin-10-deficient mice are associated with aberrant cytokine production and CD4(+) TH1-like responses. J Clin Invest.

[171] Berg DJ, Zhang J, Weinstock JV, Ismail HF, Earle KA, Alila H, Pamukcu R, Moore S, Lynch RG (2002). Rapid development of colitis in NSAID-treated IL-10-deficient mice. Gastroenterology.

[172] Hale LP, Gottfried MR, Swidsinski A (2005). Piroxicam treatment of IL-10-deficient mice enhances colonic epithelial apoptosis and mucosal exposure to intestinal bacteria. Inflamm Bowel Dis.

[173] Elliott DE, Setiawan T, Metwali A, Blum A, Urban JF, Weinstock JV (2004). *Heligmosomoides polygyrus* inhibits established colitis in IL-10-deficient mice. Eur J Immunol.

[174] Elliott DE, Metwali A, Leung J, Setiawan T, Blum AM, Ince MN, Bazzone LE, Stadecker MJ, Urban JF, Weinstock JV (2008). Colonization with *Heligmosomoides polygyrus* suppresses mucosal IL-17 production. J Immunol.

[175] Blum AM, Metwali A, Elliott DE, Berg DJ, Weinstock JV (2004). CD4+ T cells from IL-10-deficient mice transfer susceptibility to NSAID-induced Rag colitis. Am J Physiol Gastrointest Liver Physiol.

[176] Hang L, Setiawan T, Blum AM, Urban J, Stoyanoff K, Arihiro S, Reinecker HC, Weinstock JV (2010). *Heligmosomoides polygyrus* infection can inhibit colitis through direct interaction with innate immunity. J Immunol.

[177] Sutton TL, Zhao A, Madden KB, Elfrey JE, Tuft BA, Sullivan CA, Urban JF, Shea-Donohue T (2008). Anti-Inflammatory mechanisms of enteric *Heligmosomoides polygyrus* infection against trinitrobenzene sulfonic acid-induced colitis in a murine model. Infect Immun.

[178] Weng M, Huntley D, Huang IF, Foye-Jackson O, Wang L, Sarkissian A, Zhou Q, Walker WA, Cherayil BJ, Shi HN (2007). Alternatively activated macrophages in intestinal helminth infection: effects on concurrent bacterial colitis. J Immunol.

[179] Chen CC, Louie S, McCormick B, Walker WA, Shi HN (2005). Concurrent infection with an intestinal helminth parasite impairs host resistance to enteric *Citrobacter rodentium* and enhances *Citrobacter*-induced colitis in mice. Infect Immun.

[180] Chen C-C, Louie S, McCormick BA, Walker WA, Shi HN (2006). Helminth-primed dendritic cells alter the host response to enteric bacterial infection. J Immunol.

[181] Summers RW, Elliott DE, Qadir K, Urban JF, Thompson R, Weinstock JV (2003). *Trichuris suis* seems to be safe and possibly effective in the treatment of inflammatory bowel disease. Am J Gastroenterol.

[182] Summers RW, Elliott DE, Urban JF, Thompson RA, Weinstock JV (2005). *Trichuris suis* therapy for active ulcerative colitis: a randomized controlled trial. Gastroenterology.

[183] Summers RW, Elliott DE, Urban JF, Thompson R, Weinstock JV (2005). *Trichuris suis* therapy in Crohn’s disease. Gut.

[184] Saunders KA, Raine T, Cooke A, Lawrence CE (2007). Inhibition of autoimmune type 1 diabetes by gastrointestinal helminth infection. Infect Immun.

[185] Liu Q, Sundar K, Mishra PK, Mousavi G, Liu Z, Gaydo A, Alem F, Lagunoff D, Bleich D, Gause WC (2009). Helminth infection can reduce insulitis and type 1 diabetes through CD25- and IL-10-independent mechanisms. Infect Immun.

[186] Walk ST, Blum AM, Ewing SA, Weinstock JV, Young VB (2010). Alteration of the murine gut microbiota during infection with the parasitic helminth *Heligmosomoides polygyrus*. Inflamm Bowel Dis.

[187] Gillilland MG, Erb-Downward JR, Bassis CM, Shen MC, Toews GB, Young VB, Huffnagle GB (2012). Ecological succession of bacterial communities during conventionalization of germ-free mice. Appl Environ Microbiol.

[188] Ivanov II, Frutos Rde L, Manel N, Yoshinaga K, Rifkin DB, Sartor RB, Finlay BB, Littman DR (2008). Specific microbiota direct the differentiation of IL-17-producing T-helper cells in the mucosa of the small intestine. Cell Host Microbe.

[189] Ivanov, II, Atarashi K, Manel N, Brodie EL, Shima T, Karaoz U, Wei D, Goldfarb KC, Santee CA, Lynch SV, Tanoue T, Imaoka A, Itoh K, Takeda K, Umesaki Y, Honda K, Littman DR (2009) Induction of intestinal Th17 cells by segmented filamentous bacteria. Cell 139:485–498 10.1016/j.cell.2009.09.033 PMC2796826 19836068

[190] Atarashi K, Tanoue T, Shima T, Imaoka A, Kuwahara T, Momose Y, Cheng G, Yamasaki S, Saito T, Ohba Y, Taniguchi T, Takeda K, Hori S, Ivanov II, Umesaki Y, Itoh K, Honda K (2011). Induction of colonic regulatory T cells by indigenous *Clostridium* species. Science.

[191] Larsson E, Tremaroli V, Lee YS, Koren O, Nookaew I, Fricker A, Nielsen J, Ley RE, Backhed F (2012). Analysis of gut microbial regulation of host gene expression along the length of the gut and regulation of gut microbial ecology through MyD88. Gut.

[192] Weinstock JV, Elliott DE (2009). Helminths and the IBD hygiene hypothesis. Inflamm Bowel Dis.

[193] Lawrence CE, Pritchard DI (1994). Immune response profiles in responsive and non-responsive mouse strains infected with *Heligmosomoides polygyrus*. Int J Parasitol.

[194] Parker SJ, Inchley CJ (1990). *Heligmosomoides polygyrus* influence of infection on lymphocyte subpopulations in mouse mesenteric lymph nodes. Exp Parasitol.

[195] Behnke JM, Wakelin D (1977). *Nematospiroides dubius:* stimulation of acquired immunity in inbred strains of mice. J Helminthol.

[196] Wakelin D, Donachie AM (1983). Genetic control of eosinophilia. Mouse strain variation in response to antigens of parasite origin. Clin Exp Immunol.

[197] Ali NM, Behnke JM, Manger BR (1985). The pattern of peripheral blood leucocyte changes in mice infected with *Nematospiroides dubius*. J Helminthol.

[198] Ben-Smith A, Wahid FN, Lammas DA, Behnke JM (1999). The relationship between circulating and intestinal *Heligmosomoides polygyrus*-specific IgG1 and IgA and resistance to primary infection. Parasite Immunol.

